# Obituary—Prof. James McNaughton

**Published:** 1874-07

**Authors:** 


### OBITUARY.

Dr. James McNaughton, Professor of Theory and Practice of Medicine in the Albany Medical College, and President of the Faculty, died in Paris of heart disease on the 19th of June, at the age of 87. Dr. McNaughton was the oldest living teacher of medicine, having been connected with medical colleges for over fifty years. He graduated at the University of Edinburgh, Medical Department in 1816, and came to America as Surgeon to an Emigrant ship, but was persuaded to resign his position and settle in Albany, where he has since continued in the active practice of his profession. He is said not to have missed a dozen lectures in the fifty-three courses which he has delivered.

